# A systematic review and global analysis of the seasonal activity of *Phlebotomus (Paraphlebotomus) sergenti*, the primary vectors of *L*. *tropica*

**DOI:** 10.1371/journal.pntd.0010886

**Published:** 2022-12-05

**Authors:** Ahmed Karmaoui, Denis Sereno, Samir El Jaafari, Lhoussain Hajji

**Affiliations:** 1 Bioactives, Health and Environmental Laboratory, Epigenetics Team, University Moulay Ismail, Meknès, Mcorocco; 2 Faculty of Sciences and Techniques, UMI, Errachidia, Morocco; 3 Moroccan Center for Culture and Science, Zagora, Morocco; 4 IRD, University of Montpellier, InterTryp, Parasite Infectiology & Public Health Research Group, Montpellier, France; 5 IRD, CNRS, University of Montpellier MiVeGec, Montpellier, France; 6 Cluster of Competency on Health and Environment, Moulay Ismail University of Meknes, Meknès, Morocco; National Institute of Parasitic Diseases, CHINA

## Abstract

**Background:**

*Phlebotomus (Paraphlebotomus) sergenti* is a widespread proven vector of *Leishmania* pathogens causing anthroponotic cutaneous leishmaniasis (ACL), due to *L*. *tropica*, in the old world. The activity of *P*. *(Par*.*) sergenti* is seasonal and sensitive to general variations in climate. Phenological data sets can thus provide a baseline for continuing investigations on *P*. *(Par*.*) sergenti* population dynamics that may impact future leishmaniasis transmission and control scenarios.

**Methods/Principal finding:**

A systematic review of the seasonality of *P*. *(Par*.*) sergenti* was undertaken globally. Six hundred eight scientific papers were identified, and data were extracted from 35 ones, with informative data on sand fly seasonal dynamics on trapping performed from 1992 to December 2021 on 63 sites from 12 countries. Morocco, Saudi Arabia, Iraq, Iran, Pakistan, Palestine, Turkey, Spain, Portugal, Italy, Cyprus, and Georgia. The data extracted from the literature survey were further normalized. Our analysis recorded that the highest *P*.*(Par*.*) sergenti* activity occurs during the hot and dry seasons, primarily in July and August, whatever the location studied. We noticed a relationship between the latitude of sites and sand fly presence (from early April to June) and the type of density trend, varying from a single peak to multiple peaks. On a geographical scale, *P*. *(Par*.*) sergenti* concentrates between 32–37° in latitude in a large interval following the longitude and the highest number of sites with high *P*. *(Par*.*) sergenti* activity is located at the latitude 32°. We also quoted a similar seasonal dynamic and geographic distribution with *Phlebotomus (Phlebotomus) papatasi*, a proven vector of *L*. *major* that causes cutaneous infection. No apparent risk for ACL occurred from December to March, at least in the years and geographic areas considered in this survey. Altogether, knowing that high *P*. *(Par*.*) sergenti* activity would be linked with an increased risk of leishmaniasis transmission, and our study provides information that can be used for control programs on ACL transmission.

**Conclusions:**

Despite variations, we found a relatively homogeneous pattern of *P*. *(Par*.*) sergenti* potential behavior in sites whose data are published. A higher risk for *L*. *tropica* transmission was identified in the June-October period. Still, such risk was not equally distributed throughout the area since density waves of adults occurred earlier and were more frequent in some territories, like Saudi Arabia.

## Author summary

*Phlebotomus (Paraphlebotomus) sergenti* is the most widespread vector of *Leishmania* pathogens causing anthroponotic cutaneous leishmaniasis (ACL) in the old world. This paper undertook a systematic review of seasonality on a global geographical scale. This systematic analysis allowed us to identify 608 scientific papers, 35 of which 63 sites were explored in 12 countries from 1992 to December 2021. In the current review, Iran (Southern Asia) is the most influential country regarding the number of studies published on *P*. *(Par*.*) sergenti* seasonality with available data. Furthermore, the highest *P*. *(Par*.*) sergenti a*ctivity occurs in all studied locations during the hot and dry periods (with a density that peaked during the hot season, primarily in July and August). Strikingly, we recorded a similar seasonal dynamic and geographic distribution with *Phlebotomus papatasi*, a proven vector of *L*. *major* causing cutaneous leishmaniasis. On a geographical scale, sites with *P*. *(Par*.*) sergenti* activity concentrate between 32–37° in latitude in a large interval following the longitude, with the highest number of sites with high *P*. *(Par*.*) sergenti* activity is located at the latitude 32°. Altogether, knowing that high P. *(Par*.*) sergenti* activity would be linked with an increased risk of leishmaniasis transmission. Our study provides information that can be used for control programs on ACL transmission.

## Introduction

Leishmaniases rank after malaria in terms of annual incidence affecting 98 countries and territories worldwide. Visceral leishmaniasis kills between 20,000 and 30,000 persons annually; one million cutaneous leishmaniasis cases have been reported over the past five years, and over one billion people live at risk of infection. *Phlebotomus (Phlebotomus) papatasi* and *Phlebotomus (Paraphlebotomus) sergenti* are proven primary vectors of *Leishmania* parasites, *L*. *tropi*ca, and *L*. *major* respectively, causing cutaneous affection in the old world [1, 2]. Epidemiological cycles of leishmaniases can be divided into two broad categories: the zoonotic forms of leishmaniases (ZL), where the primary reservoirs are wild or domestic mammals [3], and anthroponotic forms (AL), for which humans are the primary reservoirs; *P*. *(Par*.*) sergenti* is the primary vector of the anthroponotic cutaneous leishmaniasis (ACL), and *P*. *(Par*.*) papatasi* is involved in the transmission of the zoonotic cutaneous leishmaniasis, caused by *L*. *major* (ZCL) [4]. In addition, a zoonotic cycle of *L*. *tropica* transmitted by *P*. *(Par) sergenti* is also present [5, 6] with a wide range of potential animal reservoirs [7]. The cutaneous leishmaniasis caused by *L*. *tropica* is primarily present in the Eastern Mediterranean, Middle East, North India, Afghanistan, and northeast and South Africa [8]. Besides cutaneous infections, rare visceral infections by *L*. *tropica* are documented [9, 10].

*Phlebotomus (Par*.*) sergenti*, the proven primary vector of *L*. *tropica*, is widely distributed in the old world bordering the Mediterranean area, including Morocco [11], Algeria [12], and Tunisia [13], but also in Western Asia, Saudi Arabia [14], Southern Asia, Afghanistan [12], and Iran [15], see the online website for *Leishmania* epidemiological information system (http://leishmania.ird.fr/) [16]. *Phlebotomus (Par*.*) sergenti* populations express a sizeable genetic diversity, as documented by *ITS2* rDNA and/or *Cyt b* mtDNA molecular markers and wing geometry morphometry [17]. Such variability might uncover the presence of closely related sand fly species (cryptic species [18–20]. The abundance of *Phlebotomus (Par*.*) sergenti* is primarily associated with socio-economical and biophysical factors, including precipitation [21], temperature [22, 23], and humidity [24].

The global climate changes would have an impact on the seasonal activity of *P*. *(Par*.*) sergenti*. It is, therefore, essential to gather phenological data sets to constitute a baseline for continuing investigations on *P*. *(Par*.*) sergenti* population dynamics that may impact future leishmaniasis transmission and control scenarios. To address this question, we performed a systematic review and analysis of data published on the seasonal activity of the *P*. *(Par*.*) sergenti* population on a global geographic scale.

## Material and methods

### Information sources

The selection of studies was based on searches performed (December 2021) in PubMed, Web of Science, World Wide Science, SciELO, Embase, and Google Scholar, with no specific year range and language limitation. All collected publications are listed, and the information on spatiotemporal activity and distribution of *P*. *(Par*.*) sergenti* are associated.

### Search

The search was performed using the subject headings "*Phlebotomus sergenti*", combined with several keywords, including "survey", "a monthly activity or density", "a seasonal activity or density", "leishmaniasis", "anthroponotic cutaneous leishmaniasis", “cutaneous leishmaniasis”…

### Study selection

We undertook the review following current recommendations of the PRISMA guidelines [25], taking into account the remarks for "biological" meta-analyses, which deal with non-human species [26]. First, we selected studies for inclusion in two stages. In the first stage, we screened the titles and abstracts of all citations for potentially relevant papers. In the second, we examined the full texts of these papers for the full text and data extraction. For each record, to ensure an objective assessment of all the included records, the judgments about eligibility, bias, and applicability were entirely based on the published documents and not on unpublished background information. Studies that involved the following topics were eligible for selection:

### 1-Phlebotomus sergenti

#### 2-Survey-Dynamic-record

The systematic review ruled out duplicate studies that did not include data on *P*. *(Par*.*) sergenti* presence. For the meta-analysis, data were extracted according to the following criteria: (1) Monthly data of activity, (2) Only English documents were considered, (3) For the period of studies, all years before December 2021 were considered, and (4) only peer-reviewed articles are considered and one single report. Methods of collection, such as light traps, sticky traps, or aspirators, as well as outdoor and indoor habitats, were considered. Publications, including the seasonal activity of the *P*. *(Par*.*) sergenti* attracted by blood, were not considered relevant for our systematic review. All countries or territories with data on monthly *P*. *(Par*.*) sergenti* activity are included in the study. Studies performed on multiple stations and whose results are given as the total number of trapped sand flies were considered a single site. For example, the study by Ajaoud et al., 2015 [5] in the rural area of the Azilal province was performed in three stations Ait Makhlouf II: 31°01’23”N, 6°58’53”O and Guimi: 32°00’12”N, 6°55’03”O (Beni Hassan sector), and Agmeroul: 31°58’38”N, 6°51’12”O (Tabia sector).

### Data collection process and items

We developed a data collection sheet to gather data items from studies. The data collection sheets included the following: first author name, the title and article year of publication, country, the geographic information (latitude, longitude, and altitude), period of study, the name of countries and localities were extracted, compiled, and then classified by region (South Europe, North Africa, Western Asia, and Eastern Asia).

The data was extracted from tables of the selected publications and digitized using specialized software, ‘digitizelt’ from graphs.

### Statistical analysis

Data on mensual *P*. *(Par*.*) sergenti* density were collected, with information on their geographic location. Data were extracted from the tables or digitized using the ‘digitizet’ tool (software) before being compiled by countries and regions. Due to the heterogeneity of the data collected (number of sand flies, frequencies, or incidences), the original values were normalized using Eq 1 (Eq 1) to range in a similar scale for comparison purposes. The normalized values of the vector activity of a defined month, January, for example, is the ratio between real available or extracted values (using the digitizing software) for January and the month (one month) displaying the maximal value of vector activity. This formula is applied for all collection methods to detect the monthly change and avoid heterogeneity of units.


$$Normalized\;vector\;activity\;in\;January=\frac{\left[Vector\;activity\;in\;January\right]}{\left[Month\;with\;maximal\;vector\;activity\right]})$$ Eq (1)

Values were then classified according to a scale ranging from 0 to 1, where 0 refers to the absence of activity or no data available, and 1 refers to very high activity.

### Geographic analysis

The maps were set up with QGIS 3.18.

## Results

### Study selection

**Fig 1:**
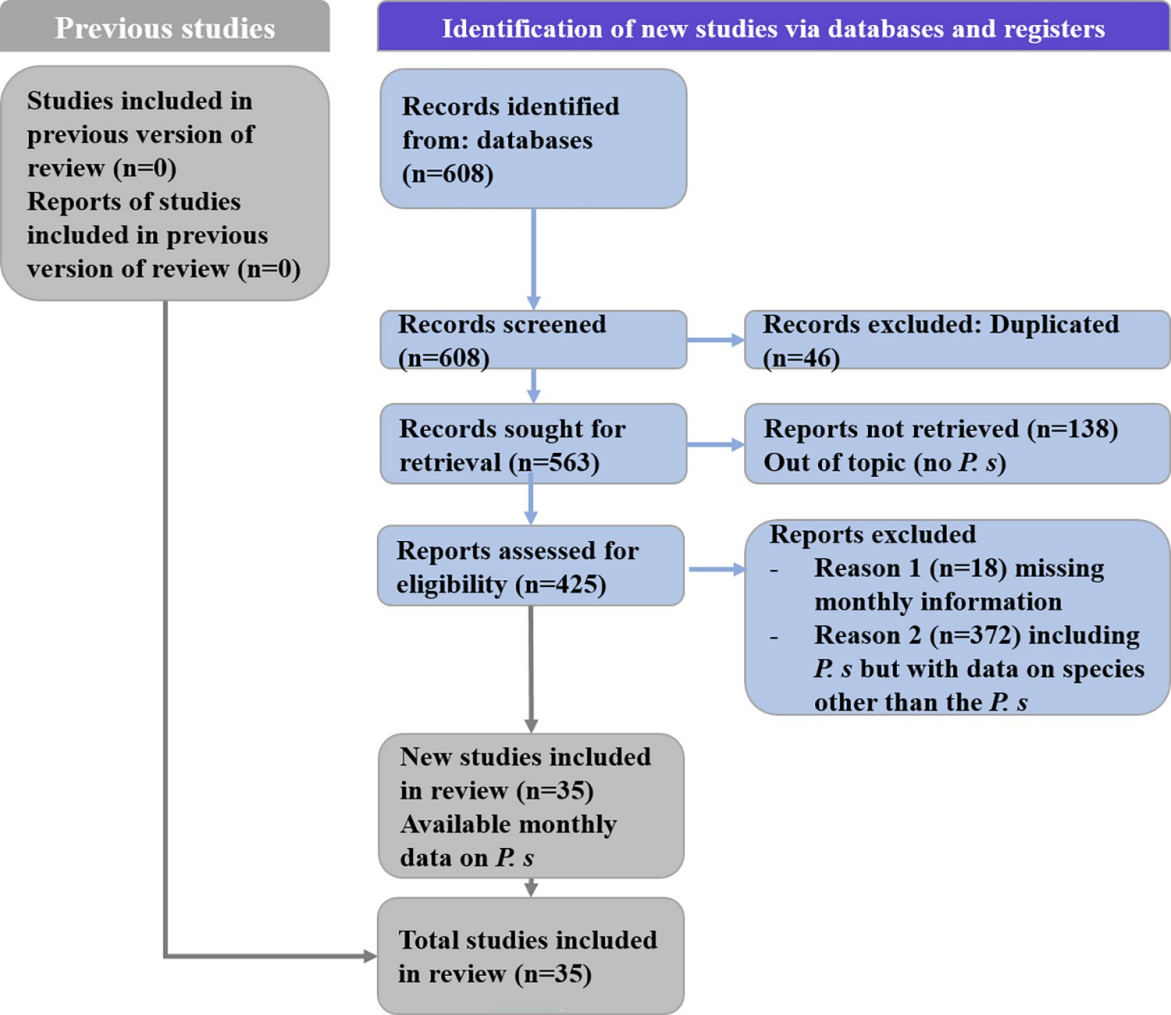
The study’s flowchart, according to PRISMA guidelines.

We collected 608 documents relevant for the analysis from PubMed, Web of Science, World Wide Science, SciELO, Embase, and Google Scholar. After removing duplicate documents (46), 562 papers were screened for relevance according to the title and abstract; 138 papers were discarded at this stage. Finally, the full text of the 425 remaining documents was carefully read, and data were extracted from 35 articles (Table 1). A diagram of the study plan, following the PRISMA statement, is given in Fig 1.

**Table 1:** Summary of the information gathered on the spatiotemporal distribution of *P*. *(Par*.*) sergenti* in Morocco, Turkey, Palestine, Saudi Arabia, Iraq, Iran, Pakistan, Spain, Portugal, Italy, Georgia, and Cyprus.

Country	N	Sites	Type of activity	Method of collection	Habitat	Latitude	Longitude	Altitude	Year	Refere-nce
Morocco	1	Rural areas of Azilal Province *	Bimodal (July and October)	Light traps,	Domestic animal shelters, Indoor	31°01’23"-32°00’12"N	6°51’12"-6°55’03"W	679–840	2011	[5]
	2	Azilal province, Ouaouizaght district	Monomodal (June)	Sticky trap night,	Stables and Indoors	32°09’27"N	6°20’57"W	~900–1200	2010	[27]
	3	Marrakech Urban	Bimodal (May and July)	Sticky traps,	Inside, around houses and stables	31°36’N	8°02’W	471	2002–2003	[28]
	4	Aichoun	Monomodal (June)	Sticky paper traps	Stables, barns, chicken, and sheep pens	33°39’N	04°38’W	~700	2011	[29]
	5	Bouasseme	Bimodal (June and September)	Adhesive traps,	Stables, barns, chicken, and sheep pens	33°31’N	04°33’W	~700	2011	[29]
	6	Sefrou	Bimodal (July and September)	Sticky traps,	Different habitats	33°39’N	04°38’W	809	2012	[30]
	7	Settat 1	Bimodal (June and September)	CDC light traps	Indoor, Holes in the dwellings’ walls, abandoned buildings, stables, and road drainage holes)	32°29’N	07°36’W	410	2014	[31]
	8	Settat 2	Bimodal (June and September)	Sticky traps	Outdoor, holes in the dwellings’ walls, abandoned buildings, stables, road drainage holes)	32°29’N	07°36’W	410	2014	[31]
Saudi Arabia	9	Al-Baha	Monomodal (May)	Light traps, aspiration, and sticky paper traps	Inside and outside houses	~20°00’N	~41°30’E	800–2500	1996–1997	[32]
	10	Mondasa, Madinah	Bimodal (May and October)	Light traps and sticky traps	Close to wall cracks, crevices, in front of animal burrows	24°46’N	39°61’E	631	2007–2008	[33]
	11	Agool, Madinah	Trimodal (June, September, and December)			24°46’N	39°61’E	631	2007–2008	[33]
Iraq	12	Tallil Air Base, Iraq	Bimodal (May and September)	Not specified	Not specified	30°56’44"N	46°05’59"E	43922	2003–2004	[23]
Iran	13	Kharameh, District, Fars province	Bimodal (June and September)	Sticky paper traps	Indoor and outdoor	29°30’4"N	53°19’15"E	1500	2014	[34]
	14	Bam city, Kerman Province	Bimodal (July and September)	Aspirated by collector	Not specified	28°16’0"N	58°7’0"E	1000–1200	2012	[35]
	15	Isfahan city 1	Bimodal (May and July)	Sticky paper traps	Indoor	32°38’N	51°29’E	1590	2005	[36]
	16	Isfahan city 2	Monomodal (August)	Sticky paper traps	Outdoor	32°38’N	51°29’E	1590	2005	[36]
	17	Outdoor, Hamadan	Monomodal (August)	Sticky traps	Outdoor	34°52’N	48°35’E	1700–1900	2013	[37]
	18	Mountainous, Qom province	Bimodal (June and September)	Sticky paper traps	Mountain	34–35°N	50–52°E	700–1800	2013	[38]
	19	Lowland, Qom provi.	Monomodal (July)	Sticky paper traps	Lowland	34–35°N	50–52°E	700–1800	2013	[38]
	20	Khorramabad (Lorestan: KL) Human dwellings	Monomodal (August)	Sticky traps	Human dwellings	~33°26’N	~48°20’E	~1100–1600	2010	[39]
	21	KL Outdoor shelters (Mountain)	Monomodal (August)	Sticky traps	Outdoor shelters	~33°26’N	~48°20’E	~1100–1600	2010	[39]
	22	KL Outdoor walls	Bimodal (August and November)	Sticky traps	Outdoor walls	~33°26’N	~48°20’E	~1100–1600	2010	[39]
	23	KL Warehouses	Monomodal (August)	Sticky traps	Warehouses	~33°26’N	~48°20’E	~1100–1600	2010	[39]
	24	Shiraz City	Monomodal (September)	Sticky traps	Indoor and outdoor	29°35’34"N	52°35’03"E	1511	2004	[40]
	25	Asalouyeh	Bimodal (April and September)	Sticky traps	Indoor and outdoor	27°28’34"N	52°36’27"E	~20–100	2009	[41]
	26	Natanz District, Isfahan	Monomodal (September)	Sticky and light traps, aspirators,	Various habitats	33°40’N	51°25’E	1600	2009	[42]
	27	Sedeh city	Bimodal (July and September)	Sticky and light traps	Indoors and outdoor	32°41’N	51° 31’E	1602	2003–2005	[43]
	28	Shiraz city	Bimodal (July and September)	Sticky and light traps	Indoors and outdoor	29°38’N	52°34’E	1486	2003–2005	[43]
	29	Mohammad Abad, Kerman (Indoor)	Monomodal (July)	Sticky paper traps	Indoor	30°14’25"N	56°36’41”E	1674	2012	[15]
	30	Mohammad Abad, Kerman (Outdoor)	Monomodal (June)	Sticky traps	Outdoor	30°14’25"N	56°36’41”E	1674	2012	[15]
	31	Ardabil Province	Monomodal (July)	sticky traps,	Indoor and outdoor	38°15’5.04"N	48°17’50.28"E		2017	[44]
	32	Sticky trap, Dehbakri (Kerman Province)	Monomodal (September)	Sticky trap	Not specified	29°3’13.89"N	57°54’37.75"E	2 052	2017–2018	[45]
	33	Sticky trap, Khajeh-Asgar (Kerman Province)	Quadrimodal (May, July, September, and November)	Sticky trap	Not specified	29°7’18.42"N	58°15’43.37"E	1 163	2017–2018	[45]
	34	Ardabil Province	Monomodal (June)	Sticky and light traps	Indoors and outdoor	37°45’–39°42’N	47.30–48.55°E		2006–2018	[46]
	35	Yazd city and its outskirts	Bimodal (April and August)	Sticky and light traps	Indoors and outdoor	31°54’N	54°15’E	581	2015	[24]
	36	Outdoor (City of Bam)	Trimodal (May, July, and September)	Light and sticky traps and/or aspiration in tubes	Outdoor	29°03’14.2"N	57°54’31.6"E	2052	2015	[22]
	37	Indoor (City of Bam)	Bimodal (May and July)	Light and sticky traps and/or aspiration in tubes	Indoor	29°03’14.2"N	57°54’31.6"E	2052	2015	[22]
Pakistan	38	Rehra (Indoor)	Bimodal (June and August)	Dog-baited sticky traps	Indoor	32°-37°N	71°E	1500–1800	1991–1992	[47]
	39	Rehra (Outdoor)	Monomodal (June)	Dog-baited sticky traps	Outdoor	32°-37°N	71°E	1500–1800	1991–1992	[47]
	40	Hudur village (Compound)	Monomodal (July)	Light traps	Compound	32°-37°N	71°E	1200	1991–1992	[47]
	41	Hudur village (Indoor)	Bimodal (May and August)	Light traps	Indoor	32°-37°N	71°E	1200	1991–1992	[47]
	42	Thor Village 1	Bimodal (May and July)	Not specified	Compound	32°-37°N	71°E	1700	1991–1992	[47]
	43	Thor Village 2	Bimodal (May and July)	Not specified	Indoor	32°-37°N	71°E	1700	1991–1992	[47]
	44	Hudur village sticky	Bimodal (May and July)	Sticky traps	Not specified	32°-37°N	71°E	1200	1991–1992	[47]
	45	Rehra village light traps(Indoor)	Bimodal (June and August)	Light traps	Indoor	32°-37°N	71°E	1500–1800	1991–1992	[47]
	46	Rehra village light traps (Compound)	Monomodal (June)	Light traps	Compound	32°-37°N	71°E	1500–1800	1991–1992	[47]
	47	Hudur village	Bimodal (August and October)	Not specified	Not specified	32°-37°N	71°E	1200	1991–1992	[47]
	48	North Waziristan Dattakhel,	Monomodal (July)	Knock-down spray catch method	Not specified	32°35’N- 33°22’N	69°22’E -70°38’E		2012	[48]
	49	North Waziristan Damdil	Monomodal (July)	Knock-down spray catch method	Not specified	32°35’N- 33°22’N	69°22’E -70°38’E		2012	[48]
	50	North Waziristan Drezanda	Monomodal (July)	Knock-down spray catch method	Not specified	32°35’N- 33°22’N	69°22’E -70°38’E		2012	[48]
Palestine	51	Jenin District	Bimodal (July and October)	Light and sticky traps and aspirators in	Various habitats	32°20’N	35°8’E	100–200	2011	[49]
Turkey	52	Cukurova region	Bimodal (June and September)	Light and sticky traps	Not specified	37°17’59"N-37°26’01"N	35°31’01"E- 35°39’27"E	150–280	2011–2012	[21]
	53	Camili village (Cukurova basin)	Bimodal (July and September)	Light, sticky, and CO2 traps, mouth aspirators, animal-baited traps, and human landing collection,	Not specified	37°20’05"N	35°36’42"E	205	2006	[50]
	54	Cukurova Basin	Bimodal (June and August)	Light traps, sticky papers, and aspirators	indoor	36°38’ 04N-37°26’54N	34°52’01E-36°28’27E	1–530	2006	[51]
Spain	55	Fuenlabrada, Madrid	Monomodal (July and August)	Light and sticky traps	Not specified	40°17’53"N	3°47’31"W	635–691	2011–2012	[21]
Portugal	56	Lisbon Metropolitan region	Monomodal (July)	Light and sticky traps,	Not specified	38°28’37"N- 38°44’51"N	9°16’52"W- 8°45’2"W	3–330	2011–2012	[21]
	57	Algarve region	Monomodal (July)			37°3’27"N- 37°14’20"N	8°37’45"W- 7°26’34"W	10–74	2011–2013	[21]
	58	Torres novas	Bimodal (June and August)	Light traps	Various habitats	39°24’–39°40’N	8°27’–8°40’W	170–600	2010	[52]
Italy	59	Urban sites of Catania, Sicily	Monomodal (July)	Sticky traps	wall holes/cavities along public roads	37°30’4"68N	15°4’27"12E	5–300	2006	[53]
	60	Urban sites of Catania, Sicily	Monomodal (July)			37°30’4"68N	15°4’27"12E	5–300	2013	[53]
Georgia	61	Vake & Mtatsminda districts, Tbilisi	Monomodal (July)	Light and sticky traps	Outdoor	41°42’07"N	44°49’31E	300–600	2006–2008	[54]
	62	Tbilisi	Monomodal (August)	Light and sticky traps	Not specified	41°42’01"N- 41°44’08"N	44°48’59"E- 44°49’30"E	495–603	2011–2013	[21]
Cyprus	63	Steni	Bimodal (July and September)	Light and sticky traps	Not specified	34°59’54"N	32°28’17"E	200	2011–2012	[21]

~ altitude deducedo from coordinate references using Google Earth

* Three places were considered in one site called rural areas of Azilal province: A total of 4,407 sand flies were collected in the three rural places of Azilal province.

### A systematic review of *P*. *sergent*i seasonal activity

**Fig 2:**
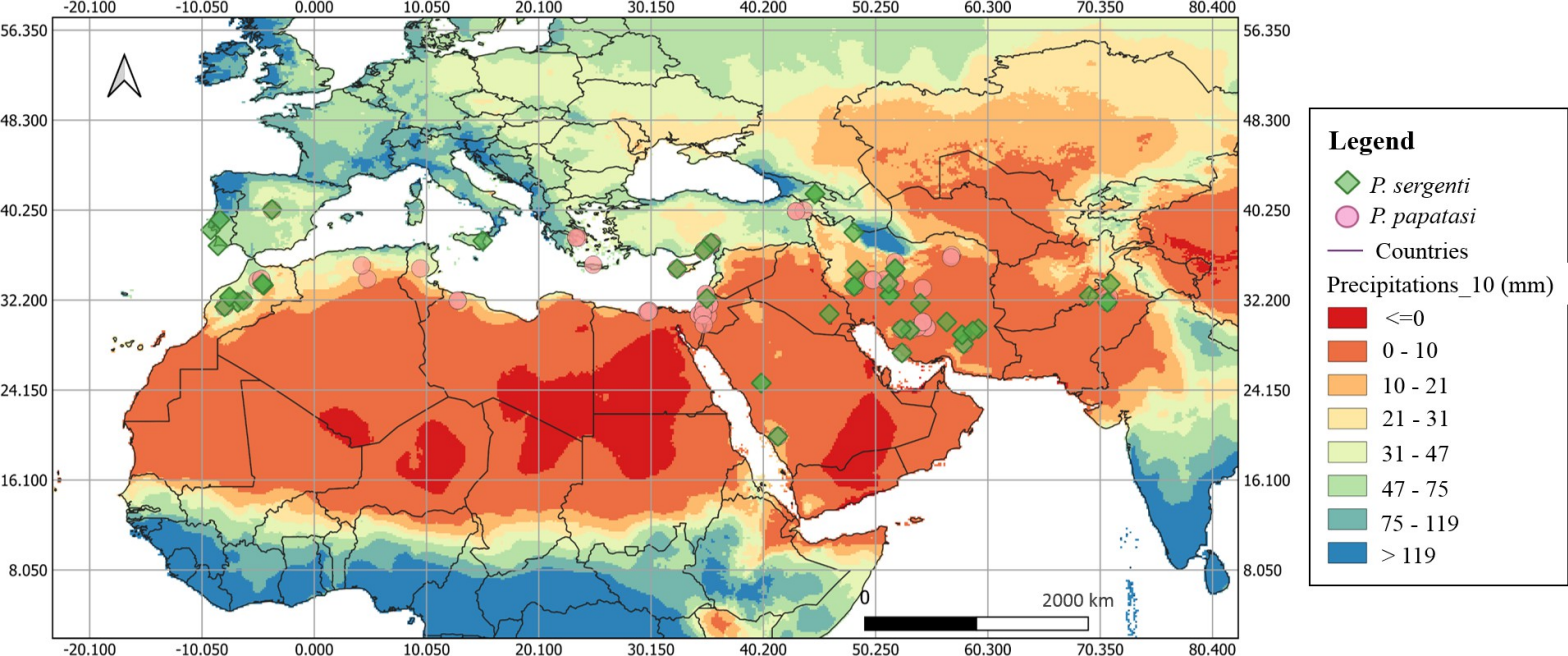
The geographic location where data on *P*. *(Par*.*) sergenti* are published. Sites with data on the seasonal density of *P (Par*.*)*. *sergenti* and the annual precipitation (mm). *Phlebotomus* (*Phlebotomus*) *papatasi* geographic distribution data were extracted in a previously published work [55]. Precipitation data were collected at ESRI grids, resolution 10 min according to WorldClim 1.4 (current conditions), http://worldclim.com/current; [56]). Boundaries were adapted and modified from Open Data Soft https://public.opendatasoft.com/explore/dataset/world-administrative-boundaries/export/.

Available monthly data *P*. *(Par*.*) sergenti* activity can be extracted for seventy locations from 12 countries representing three regions: the North African region including Morocco, the Sothern Europe that represents Spain, Portugal, Italy, Georgia, and Cyprus; the Asian region by Turkey, Palestine, Saudi Arabia, Iraq, Iran, and Pakistan. The information on locations extracted is represented in Fig 2 and Table 1. Interestingly similar geographic distribution with *Phlebotomus papatasi*, another vector of a pathogen causing cutaneous leishmaniasis (*L*. *major*), is observed (Fig 2). A previous meta-analysis found a similar global geographic distribution of *P*. *papatasi* [55] for *P*. *sergenti*. This may indicate that most studies dealing with *P*. *papatasi* have also identified the presence of *P*. *sergenti* and other sandfly species.

### Europe

**Fig 3:**
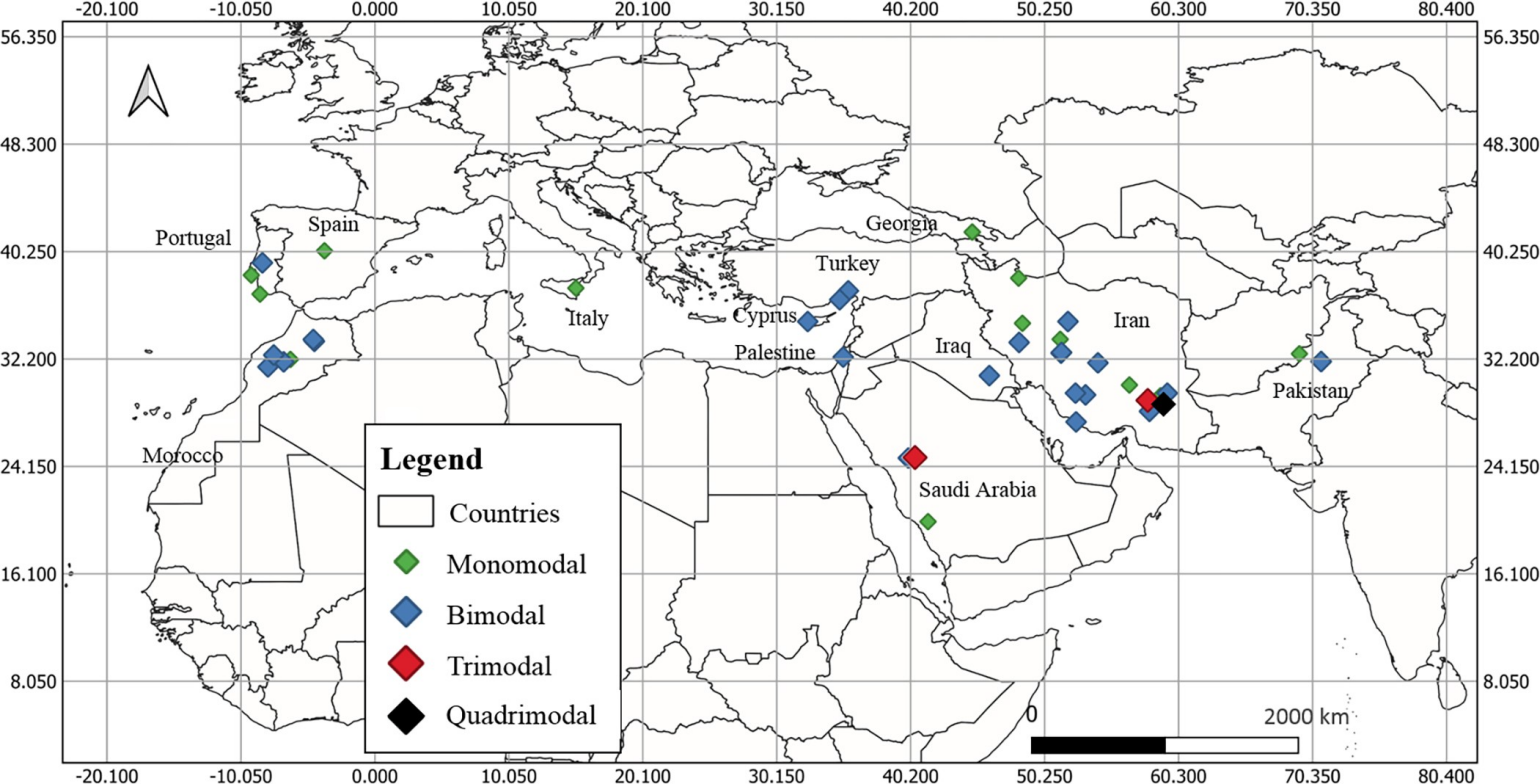
According to data collected on seasonal activity investigation, the geographic distribution of *P*. *(Par*.*) sergenti*. A Mono-modal, bimodal or trimodal distribution of the normalized sand fly activity has been recorded from data extracted from 35 published scientific works. Boundaries were adapted and modified from Open Data Soft https://public.opendatasoft.com/explore/dataset/world-administrative-boundaries/export/.

**Fig 4:**
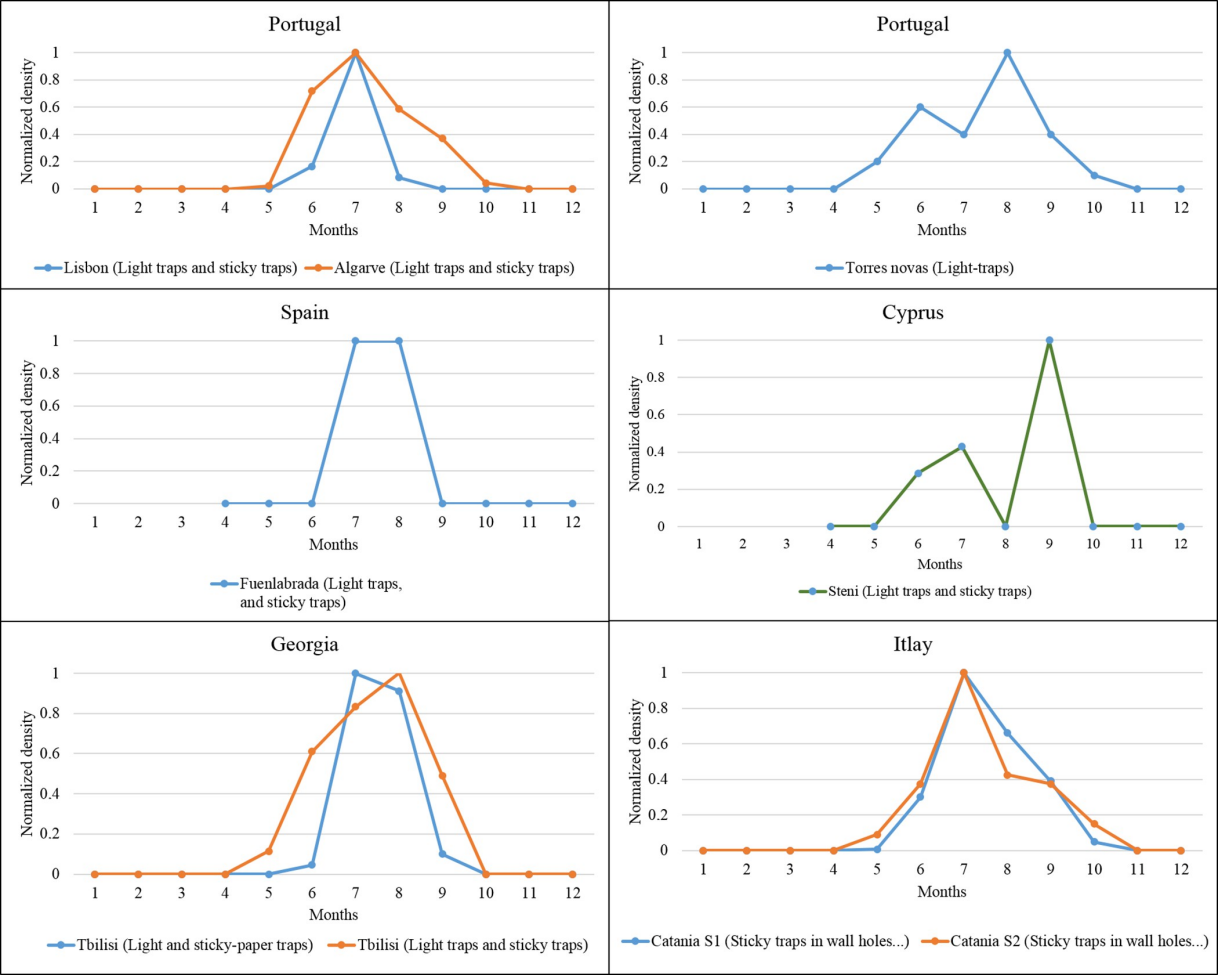
Seasonal *P*. *(Par*.*) sergenti* density (normalized values) recorded in southern European countries (Spain, Italy, Portugal, Cyprus) and Georgia as revealed by our meta-analysis Data collected from different studies performed in the same locality are grouped in the same figure.

Monomodal and bimodal seasonal density distribution are recorded at the European scale in countries where data are available. In Portugal, a monomodal seasonal distribution (with the peak in July) is recorded in the regions of Algarve and Lisbon, and bimodal density distribution with the highest density in June and August in Torres nova (Figs 3 and 4). A single-density peak is recorded in Madrid, Spain, increasing activity from July to August. In Georgia, for data collected in 2012–2013, the *P*. *(Par*.*) sergenti* density was maximal in July, and for data gathered in 2006–2008, in August. A bimodal distribution with tops in July and September is found regarding Cyprus. In Italia, a monomodal distribution, top in July, is recorded in two prospected sites of Catania. So, from the data we have collected, July would be the month with the higher risk of anthroponotic leishmaniasis in European countries.

### Africa, North Africa

**Fig 5:**
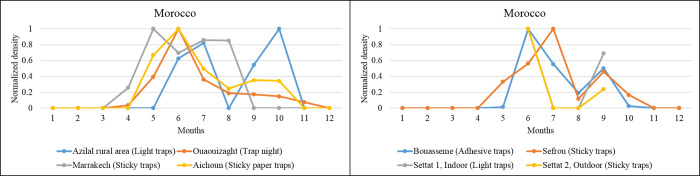
Seasonal *P*. *(Par*.*) sergenti* density (normalized values) extracted for Morocco, North Africa, as revealed by our meta-analysis. Data collected from different studies performed in the same locality are grouped in the same figure. Asia, western Asia. Turkey’s bimodal sand fly density distributions are recorded (Figs 3 and 6 and Table 1). In the Curkova area, two series of data collected in 2007 and from 2011 to 2012 were extracted from the literature. Both display a bimodal distribution of *P*. *(Par*.*) sergenti* density, with maximum peaks occurring in June, July, and September.

A bimodal distribution of the *P*. *(Par*.*) sergenti* density is evidenced in Morocco, with maximal densities varying between stations. For example, in Azilal rural area, a bimodal distribution with higher densities in July and October is registered. In Marrakech, peaks occur in May and July, Aichoun and Bouassemein in June and September, and Sefrou in July and September. In Ouaouizaght, a monomodal distribution is recorded as a bimodal activity trend (Fig 5). Overall, for three stations, peak density occurs in September and July; for two stations, another peak occurs in June; in Morocco, the period at risk of transmission is in July and September in most of the stations where data are available.

**Fig 6:**
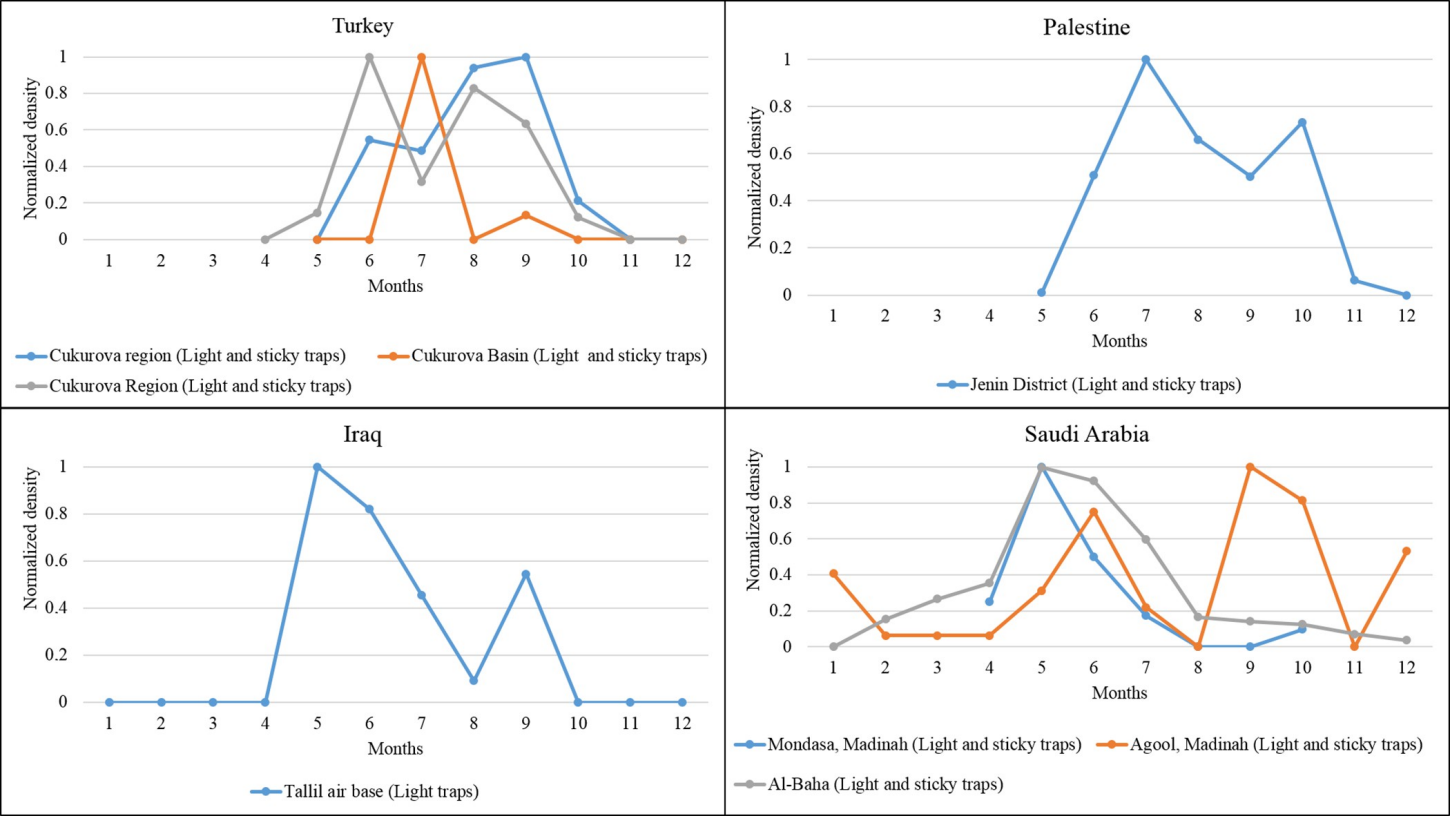
Seasonal *P*. *(Par*.*) sergenti density* (normalized values) extracted for western Asian countries (Turkey, Iraq, Saudi Arabia, Palestine), as revealed by our meta-analysis. Data collected from different studies performed in the same locality are grouped in the same figure. *Southern Asia*. Data on the seasonal activity of *P*. *(Par*.*) sergenti* were gathered from various geographic areas with indoor or outdoor sampling (Fig 7).

In Palestine, a bimodal distribution is also recorded in the Jenin District, with the highest activity in July and October (Fig 6).

In Saudi Arabia, a bimodal distribution of the seasonal *P*. *(Par*.*) sergenti* density was recorded in Mondasa, with the highest density in May, while a monomodal distribution was found in Al-Baha (Fig 6). On the other hand, in Agool, a trimodal distribution is recorded with high-density peaks in June, September, and December. Therefore, in Saudi Arabia, in Agool, the risk of transmission occurs primarily from May to June, September, and December (Fig 6).

In Iraq, only one study was found during our literature survey. However, the data extracted depicts a bimodal distribution with the highest density in May and September (Fig 6).

According to all these data, in western Asia and most countries from which data are published, the period at risk of transmission spans Mai to September and December in Saudi Arabia.

**Fig 7:**
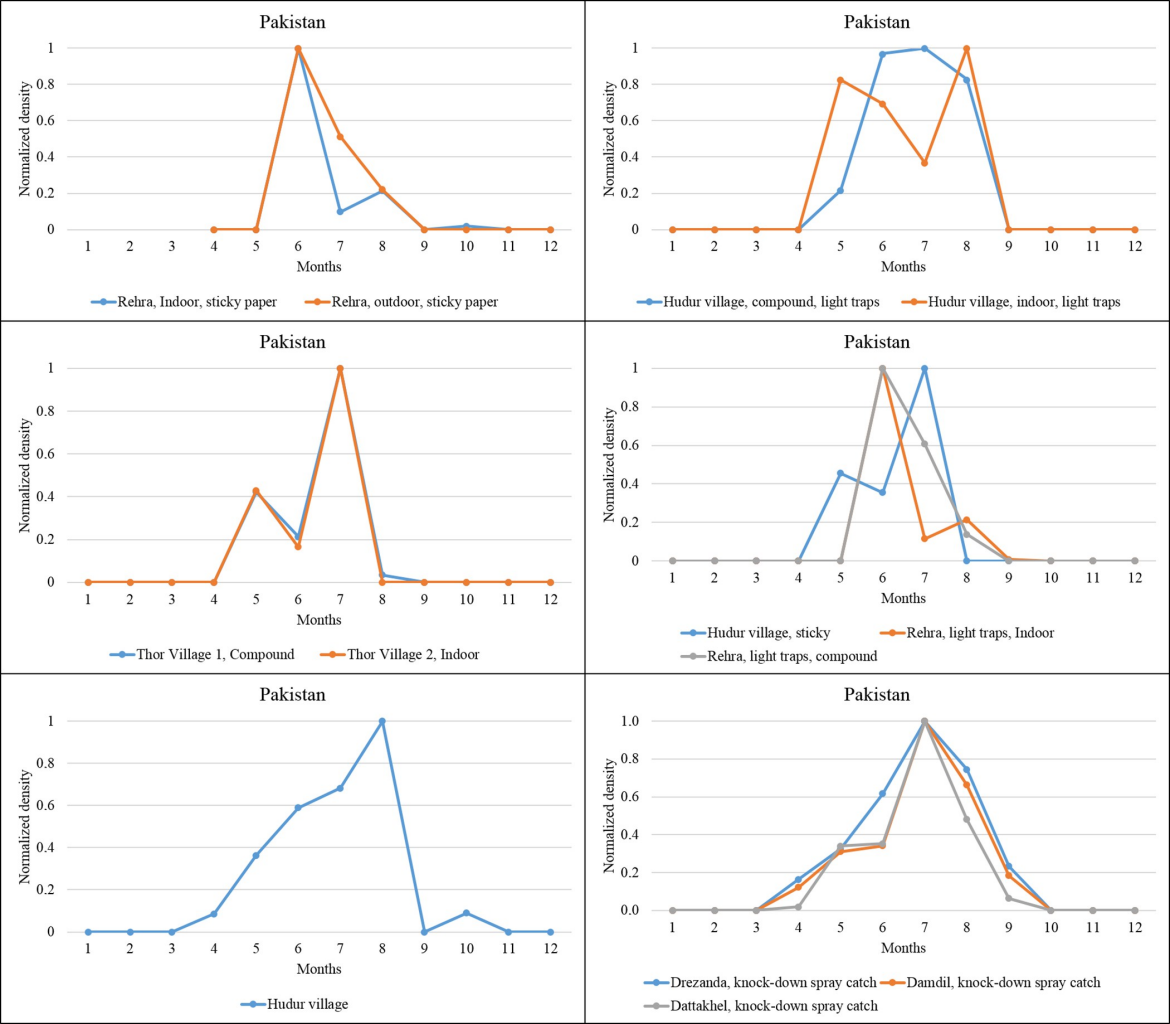
The seasonal *P*. *(Par*.*) sergenti* density (normalized values) extracted for southern Asia countries. Sites from the same locality were gathered in a single graph.

In Pakistan, in the Rehra locality, a study with indoor sticky papers disclosed a bimodal distribution, with higher density in June and August. A monomodal distribution, with a peak of density in June, is recorded when a monomodal distribution is recorded using outdoor sticky traps (Fig 7). However, in the village of Hundur, data collected with light traps disclosed a monomodal distribution of the activity with a peak of density in July, while in outdoor sites, a bimodal distribution of the density is recorded in May and August. However, the village of Thor recorded a similar seasonal distribution of *P*. *(Par*.*) sergenti* activity (Fig 7). The other stations of Hundur sampled with sticky paper display a bimodal distribution of the activity in May and July. The two sites in Rehra using light traps depict a bimodal distribution for indoor traping. Another village at the site of Hundur recorded monomodal activity with a peak in August (Fig 7). While the three areas from North Waziristan (Drezanda, Damdil, and Dattakhel) recorded a monomodal distribution with maximum activity in July when using knock-down spray catch. Four stations recorded a density peak in June, five in July, and two in August, indicating a high risk of *Leishmania* transmission in June and July.

**Fig 8:**
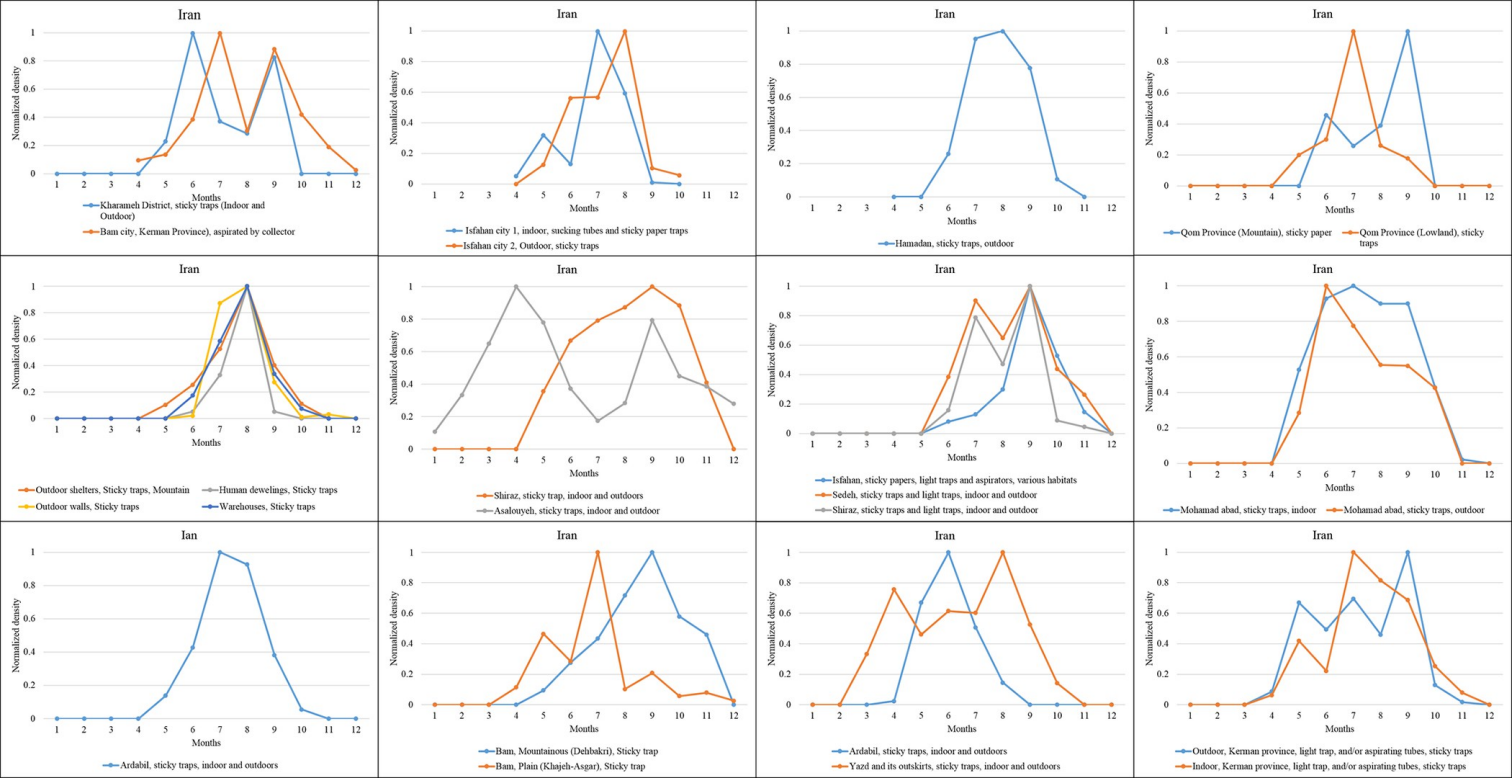
The seasonal density of *P*. *(Par*.*) sergenti* (normalized values) was extracted from data collected for Iran.

The data collected highlight that many studies on *P*. *(Par*.*) sergenti* seasonal dynamic were performed in Iran (Southern Asia, Fig 8). In Kharameh district and Bam city, seasonal dynamics follow a bimodal distribution, with the maximum density in September, June, or July. In Isfahan city, the data collected from indoor trapping displays a bimodal distribution (in May and July). A monomodal distribution was ascertained when the survey was outdoors, with a single peak in August indoors recorded. A monomodal seasonal distribution was also recorded in the city of Hamadan (west of Iran), with the highest density in August. In the province of Qom, a bimodal density dynamic (June and September) is recorded in the mountain area, while a single peak (July) occurs in the lowland. In Khorramabad, a mountainous area, trapping was performed in four stations, outdoor shelters, Human dwellings, outdoor walls, and warehouses. A monomodal distribution with the highest density in August is recorded in all these locations. In addition, using indoor and outdoor sticky papers, a monomodal distribution is recorded in Shiraz, with a peak in September. While in Asalouyeh, a bimodal distribution was recorded with maximum activity in April and September. In Isfahan, a monomodal vector distribution was found in various habitats and combined many trapping methods such as sticky, papers, light traps, and aspirators. However, a bimodal activity was recorded in both Sedeh and Shiraz stations, with two peaks recorded in July and September indoors and outdoors using sticky traps and light traps. A monomodal distribution was reported in Mohamad Abad indoor sites, with a density peak in July, while in outdoor places, the density peaks in June. A monomodal distribution was reported in Ardabil after indoor and outdoor sampling of sandflies using sticky papers. The maximal activity was recorded in July (Fig 8). The same modality was also recorded in the Bam mountainous area of Dehbakri, with a peak in September. A quadrimodal repartition was reported in the plain area of Khajeh-Asgar, with peaks in May, July, September, and November. In the second study in Ardabil and using indoor and outdoor sticky traps, monomodal repartition with a maximum in June is registered. However, a bimodal distribution in Yazd and its outskirts is recorded with peaks in April and August when sampling was performed indoors and outdoors with sticky papers. In the indoor habitat of the Kerman province, with light trap, and/or aspirating tubes, and sticky traps, a bimodal activity was found with maximums in May and July, while a trimodal distribution was reported outdoors, with peaks in May, July, and September (Fig 8).

### General overview *of P*. *(Par*.*) sergenti* seasonal activity

**Fig 9:**
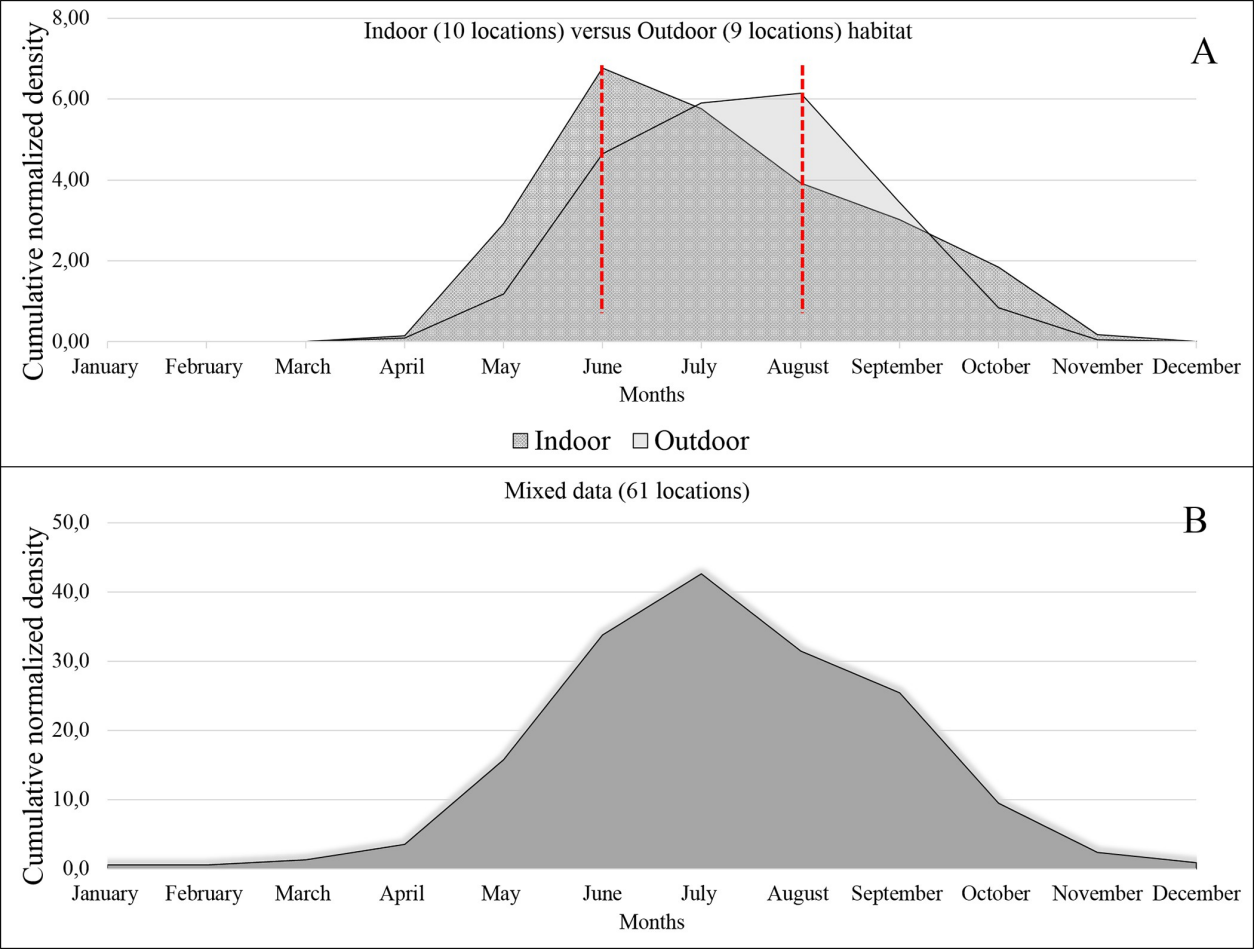
Cumulative normalized seasonal density of *P*. *(Par*.*) sergenti* in the 12 countries included in the study. Normalized densities were pooled from the 63 locations in 12 countries collected in various habitats (indoor-outdoor) using a panel of trapping methods (Light, sticky, or CO2 traps, aspirator…) (A) Normalized densities were pooled from 10 locations in countries (Morocco, Pakistan, Iran, and Georgia) indoor habitat and 9 locations in 3 countries (Morocco, Pakistan, Iran) for outdoor habitat; (B) Mixed data (Indoor and outdoor) of the 63 locations.

Our global geographic scale analysis can conclude that within sites whose data are available, eight present a high density in September, Six in August and July, and two in June. Therefore, September might be the month with the highest risk of transmitting *Leishmania* agent in that country, followed by August and July. We then aggregate data from the various documented sites to draw a graph depicting the global dynamics in the 12 countries included in the meta-analysis (Fig 9). Although these data are heterogeneous, they provide information on the transmission risk of *L*. *tropica* in link with *P*. *(Par*.*) sergenti* dynamic at a global geographic scale. Notably, the cumulative monthly density data discloses that sand flies’ emergence occurs with a density peak in July, with a relatively high density of *P*. *(Par*.*) sergenti* present from June to September (Fig 9A). Remarkably, in locations where trapping was performed in indoor habitat, the density peak appears 2 months earlier than in places whose trapping was performed outdoors (Fig 9A). Nevertheless, the emergence period is similar for both habitats, primarily in April (Fig 9A and 9B). Then *P*. *(Par*.*) sergenti* density rapidly decreases after September, diapause being present in all locations studied probably after December.

Then the potential relationship between season (months) and the latitude, with a high score for vector abundance, was investigated. First, the month with the highest relative vector density was summed for the 63 locations and reported as a latitude function (Table 2). The findings show that the latitudes 32, 29, and 37 N° recorded most areas with a high abundance of this vector. At the same time, July (29) is the month with the highest locations recording a high quantity of *P*. *(Par*.*) sergenti*, followed by August (19), June (17), and September (15).

**Table 2:** Seasonal *P*. *(Par*.*) sergenti* density according to the latitude. The month with the highest relative density in the 63 locations was reported as a function of the latitude.

Latitude °	April	May	June	July	August	September	October	Total
20		1	1				0	2
24		1				1	1	3
27	1							1
28				1		1		2
29			1	2	1	5	1	10
30		3	3	1	1	1		9
31		1		2	2		1	6
32		1	8	10	3	1		23
33			2	2	4	1		9
34				2	1	2		5
36			1		1			2
37			1	4	1	2		10
38				2	1			3
39					1			1
40				1	1			2
41				2	2			4
Total	1	7	17	29	19	15	3	

**Fig 10:**
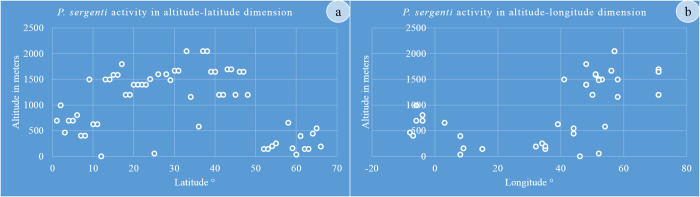
*P*. *(Par*.*) sergenti* seasonal activity according to (a) Altitude- Latitude and (b), Altitude-Longitude.

The analysis of our dataset discloses an association between the geographic distribution of *P*. *(Par*.*) sergenti* and the elevation (Fig 10). The high altitude areas primarily concentrate sites from 40 to the 70E° and those of lower altitudes below 10E° longitude. On the other hand, when considering the latitudes, sites with high elevations localized mainly between the 10 and 50N°of latitude, those of lower altitudes are found above 50 N° and below 10N°.

## Discussion

A probable rapid global average temperature rise with earlier shifts and increased magnitude is expected in the near future [57]. While this phenomenon has already caused the geographical expansion of several arthropod-borne diseases, it is likely to affect temporal parameters of seasonally transmitted infections such as leishmaniasis. The activity period of adult sand flies is typically seasonal. Because seasonal phenomena are sensitive to temperature, phenological observations would shed light on ongoing climate changes and geographical dispersion parameters. Anthroponotic cutaneous leishmaniasis caused by *L*. *tropica* and transmitted primarily by *P*. *(Par*.*) sergenti* is considered a significant public health issue with dramatic psychosocial consequences and expansion into previously non-endemic zones [31]. The capacity of *P*. *(Par*.*) sergenti* to inhabit extremely diverse ecoregions has the potential for vast geographical dispersion. Knowing that the spread of ACL to new foci is likely linked to human travel [58], [4]. The significant impacts of anthropogenic climate change on human migration [59] would also facilitate the dissemination of ACL into previously ACL-free areas.

To address risks of seasonal transmission and regional expansion of ACL, knowledge of *P*. *(Par*.*) sergenti* dynamics would be required as a starting baseline for continuing investigations on changes that may impact leishmaniasis transmission. This systematic review intends to address this baseline questioning.

Knowledge of the seasonal dynamic of arthropod vectors is of paramount importance for vector-borne transmitted diseases like leishmaniasis. Therefore, we collected several studies depicting the seasonal activity of *P*. *(Par*.*) sergenti*. All these studies disclose that seasonal abundance of *P*. *(Par*.*) sergenti* followed mono, bi, or trimodal distribution. Furthermore, the systematic review confirms that temperature is a significant determinant for the activity start of *P*. *(Par*.*) sergenti*. During cold months sand flies undergo diapause as the fourth larval stage (L4), and after a rise in temperature to a certain level, L4 larvae pupate, and adults emerge within two or three weeks. Thus, pupation and adult emergence periods in sites were probably dependent on generalized temperature increase to which L4 larvae were exposed in their natural environment.

Interestingly, data extracted from the literature *for P*. *(Par*.*) sergenti* depicted a quite similar geographical dispersion with those *P*. *(Phl*.*) papatasi*, with high activity of these vectors between the latitudes 30–40° as well as a significant presence in the longitude dimension [55]. *Phlebotomus (Par*.*) sergenti* is considered a “mountainous” vector [60], but Doha & Samy, [32] doesn’t report the effect of the elevation on the presence of *P*. *(Par*.*) sergenti*. In addition, Büttiker & Lewis [61] reported higher density at low heights, 500–700 m. None or few *P (Par*.*)*. *sergenti* are trapped in plains and coastal zone [62], but *P*. *(Par*.*) sergenti* is present in urban areas of Shiraz [40], Isfahan [36], and Marrakech [28]. The presence of *P*. *(Phl*.*) papatasi* was abundant at 0–200 m and 800–1200 m elevation. Our analysis of collected data points that at altitudes>1000 meters, sites with high vector abundance localized mainly between 40 to 70E° and in areas with elevation >600 meters below 10E°. Most sites with >1000 meters of elevation are between 10 and 50N°, and places with elevation <1000 meters are above 50 N° and below 10N°. The fact that altitudes, latitudes, and longitudes correlate with the vector distribution would indicate the impact of environmental factors on vector abundance and ACL incidence. The local environment impact ACL incidence, as explored in the Herat province in Afghanistan, using logistic regression modeling [63]. At the local scale, soil types, land cover, altitude, and proximity to a river impact the incidence of ACL. The higher humidity provided by irrigated areas and the moist soil at altitudes ranging from 700 to 1200 m offer suitable breeding conditions for sand flies. Climatic variables like humidity, precipitation, and temperature directly affect vector density. For example, higher *P*. *(Phl*.*) papatasi* abundance is between 28 to 34° C and 31 to 33° for *P*. *(Par*.*) sergenti* [64]. For both vectors, the maximum density is recorded at 20:00 h, and the minimum was at 06:00 hour in the September period 18:00–06:00 hour [40].

Our literature survey gathers data using various sampling methods that are Light traps (LT) and sticky traps (ST) [48], CO_2_ traps, mouth aspirators, human landing collection, animal-baited traps [50], and the Knockdown spray catch method [48]. In our survey, only data from sticky paper (used in 47 sites) and light traps (used in 25 sites) were considered. Other methods include aspirators, CO_2_ traps, and Knockdown spray catch methods that have been only marginally used.

In conclusion, we collected and analyzed patchy data dispersed in time and space, which includes mostly one season, mainly in a single site at different periods. Since seasonal phenomena are susceptible to variations in temperature, any conclusion on the phenological observations we collected and aggregated must be taken with care. Nevertheless, we reported that systematic review and extraction of data for normalized seasonal activity analysis at a large geographical scale could provide information on patterns of the potential behavior of ACL vectors, guiding the choice of sentinel areas for future monitoring. Hence, recent and accurate knowledge of *P*. *(Par*.*) sergenti* dynamics would be required as a starting baseline for continuing investigations on changes that may impact ACL incidence.
